# Metatranscriptomic and metataxonomic insights into the ultra-small microbiome of the Korean fermented vegetable, kimchi

**DOI:** 10.3389/fmicb.2022.1026513

**Published:** 2022-10-05

**Authors:** Hae-Won Lee, So-Ra Yoon, Yun-Mi Dang, Ji-Hyun Yun, Hoibin Jeong, Kil-Nam Kim, Jin-Woo Bae, Ji-Hyoung Ha

**Affiliations:** ^1^Hygienic Safety Packaging Research Group, World Institute of Kimchi, Gwangju, South Korea; ^2^Department of Biology, Kyung Hee University, Seoul, South Korea; ^3^Department of Life and Nanopharmaceutical Sciences, Kyung Hee University, Seoul, South Korea; ^4^Chuncheon Center, Korea Basic Science Institute (KBSI), Chuncheon, South Korea; ^5^Korea Research Institute of Bioscience and Biotechnology (KRIBB), Daejeon, South Korea

**Keywords:** metatranscriptome, metataxonome, tangential flow filtration, ultra-small microbiome, kimchi

## Abstract

Presently, pertinent information on the ultra-small microbiome (USM) in fermented vegetables is still lacking. This study analyzed the metatranscriptome and metataxonome for the USM of kimchi. Tangential flow filtration was used to obtain a USM with a size of 0.2 μm or less from kimchi. The microbial diversity in the USM was compared with that of the normal microbiome (NM). Alpha diversity was higher in the USM than in NM, and the diversity of bacterial members of the NM was higher than that of the USM. At the phylum level, both USM and NM were dominated by *Firmicutes.* At the genus level, the USM and NM were dominated by *Lactobacillus*, *Leuconostoc*, and *Weissella,* belonging to lactic acid bacteria. However, as alpha diversity is higher in the USM than in the NM, the genus *Akkermansia*, belonging to the phylum *Verrucomicrobia*, was detected only in the USM. Compared to the NM, the USM showed a relatively higher ratio of transcripts related to “protein metabolism,” and the USM was suspected to be involved with the viable-but-nonculturable (VBNC) state. When comparing the sub-transcripts related to the “cell wall and capsule” of USM and NM, USM showed a proportion of transcripts suspected of being VBNC. In addition, the RNA virome was also identified, and both the USM and NM were confirmed to be dominated by *pepper mild mottle virus* (PMMoV). Additionally, the correlation between metataxonome and metatranscriptome identified USM and NM was estimated, however, only limited correlations between metataxonome and metatranscriptome were estimated. This study provided insights into the relationship between the potential metabolic activities of the USM of kimchi and the NM.

## Introduction

Kimchi is not only a representative food of Korea (Kim et al., 2016) but is one that has been consumed in Korea for the past 2000 years (Kwon et al., 2014; Park et al., 2014). Kimchi, of which there are approximately 200 types is a salted fermented vegetable made by first salting kimchi cabbage (*Brassica rapa* subsp. *pekinensis*), mixing it with condiments such as red pepper powder, onion, garlic, and ginger, and then allowing it to ferment. Kimchi is, therefore, considered unique in its manufacturing method compared to other fermented-salted vegetables that are primarily pickled in salt or vinegar. Further, kimchi has been reported to be a good source of probiotics containing lactic acid bacteria (Song and Lee, 2014) and has been suggested as a functional food with low calories that is rich in vitamins and minerals as well as dietary fiber (Kim et al., 2016). Recently, during the coronavirus pandemic, the antiviral effects of kimchi became known (Bousquet et al., 2021; Das et al., 2021), and kimchi, which was mainly consumed in East Asia, is now being consumed worldwide (Lee and Ko, 2021).

The shotgun-based high throughput sequencing approach provides an opportunity to map the microbiome of foods to an unprecedented depth, highlighting the importance and key factors shaping the composition and activity of the resident microbiome, influencing food quality and safety (De Filippis et al., 2021; Quijada et al., 2022). An example is metatranscriptome analysis, which interprets RNA transcripts by high-throughput sequencing and offers unprecedented opportunities to analyze the functional and taxonomic dynamics of a microbiome (Bikel et al., 2015; Gallardo-Becerra et al., 2020).

Since ultramicrobacteria (UMB) were first defined by Torrella and Morita (1981), they have been identified in various environments such as soil, sand, ice, lake water, and seawater (Nakai, 2020), and an ultra-small microbiome (USM) containing UMB has been identified (Cai et al., 2015; Ghuneim et al., 2018; Proctor et al., 2018; Liu et al., 2019). In this study, USM is a microbiome containing ultra-small size microorganisms such as bacteria, endospores, and filterable bacteria, whose size has been reduced by external extreme environmental conditions, as well as UMB. The USM has been defined fairly comprehensively as a microbiome that passed through a 0.2 μm pore size filter.

In our previous study (Lee et al., 2022), although there was little possibility of expression, traces of pathogens remained in USM. It was thought to be possible that judging from this, some bacteria such as VBNC, persisters, spore forms, and filterable forms in USM may act as potential microbiological hazards in fermented foods, whereas, if there are previously unknown health benefits of USM, it may be possible to make a unique product by fractionating and concentrating it. Although this USM may be important in food, little research on it is currently being done. Moreover, as far as we know, no metatranscriptome analysis has been attempted on the USM of fermented foods.

Our group recently confirmed the presence of communities of UMB of size 0.2 μm or less in fermented cabbages such as kimchi and sauerkraut using tangential flow filtration (TFF) and 16S rRNA gene amplicon sequencing (Lee et al., 2022). It was also revealed that the diversity of ultra-small bacterial microbiomes smaller than 0.2 μm in fermented cabbages was high. However, that study could not confirm the bacterial microbiome and metabolic activities. Therefore, in this study, we analyzed the metatranscriptome and metataxonome for the USM of kimchi. We also tried to gain insights into the relationship between the potential metabolic activities of the USM of kimchi and the microbiome.

## Materials and methods

### Sample preparation

For this study, kimchi made in South Korea was purchased from an online market in October 2021. The main ingredient of kimchi is kimchi cabbage (*Brassica rapa* subsp. *pekinensis*), and 10 packets of kimchi of 400 g each (to make handling easier) were purchased. After purchase, the pH of kimchi was measured with an automatic pH/mV titrator (TitroLine 5000, SI Analytics, Germany). All the kimchi samples were merged in a double-structured basket sterilized with a built-in strainer, and broths from merged kimchi samples were obtained and roughly filtered by the strainer. Then, 500 ml of Dulbecco’s phosphate buffered saline (DPBS, Welgene, Korea) was added to the basket to facilitate filtration. The work on sample preparation was done inside a clean bench.

### Pre-filtration and tangential flow filtration

Pre-filtration and TFF were performed similarly to the previous study (Lee et al., 2022). The broth obtained from the kimchi samples was pre-filtered by a polypropylene capsule filter (GVS Filter Technology, United States) with a pore size of 10 μm for TFF. Pre-filtration was carried out smoothly using a vacuum pump inside a clean bench.

Pre-filtrated kimchi broth was injected into a TFF system (Cogent μScale TFF System, Millipore, United States), and TFF was performed in two phases. In the first phase, a 0.22 μm pore size TFF cartridge (Pellicon 2 Mini Cassette, Media: Durapore 0.22 μm, Millipore, United States) was used to confirm a normal microbiome (NM) larger than 0.2 um in size. Then the NM was concentrated to 20 ml, and after dividing by each 10 ml, 25 ml of RNAlater (Sigma, United States) for metatranscriptomic analysis was separately added to 10 ml of concentrate for stabilization and protection of RNA. The remaining 10 ml was used for metataxonomic analysis. The prepared NM samples were stored in a −20°C freezer until analysis. A 100 kDa molecular weight cut-off (MWCO) TFF cartridge (Pellicon 2 Mini Cassette, Media: Biomax 100 kDa, Millipore, United States) was used in the second phase to confirm a USM smaller than 0.2 μm in size. Then the USM was concentrated to 20 ml, and after dividing by each 10 ml, 25 ml of RNAlater (Sigma, United States) for metatranscriptomic analysis was separately added to 10 ml of concentrate for stabilization and protection of RNA. The remaining 10 ml was used for metataxonomic analysis. The prepared USM samples were stored in a −20°C freezer until analysis.

### Metataxonomic analysis

For metataxonomic analysis based on the 16S rRNA gene, DNAs were extracted using a DNeasy PowerSoil kit (Qiagen, Germany) for each sample solution of NM and USM. The extracted DNAs were quantified using the Quant-IT PicoGreen assay kit (Invitrogen, United Kingdom) following the manufacturer’s instructions. The quantified nucleic acids were amplified for the V3 and V4 regions of the 16S rRNA gene by a universal primer pair with Illumina adapters (V3-F, 5′-TCGTCGGCAGCGTCAGATGTGTATAAGAGACAGCCTACGGGNGGCWGCAG-3′; V4-R, 5′-GTCTCGTGGGCTCGGAGATGTGTATAAGAGACAGGACTACHVGGGTATCTAATCC-3′). The polymerase chain reaction (PCR) conditions were as follows: initial denaturation at 94°C for 3 min, followed by 25 cycles of denaturation at 95°C for 30 s, then annealing at 55°C for 30 s, then extension at 72°C for 30 s, and a final extension at 72°C for 5 min. The PCR products were purified using AMPure beads (Agencourt Bioscience, United States). The purified PCR products were re-amplified to 15 cycles, including initial denaturation and final extension steps, as described with the Nextera XT indexed primer pair. The re-amplified PCR products were re-purified using AMPure beads and were quantified using the KAPA Library Quantification kits for Illumine sequencing platforms (Kapa Biosystems, United States). The qualification was carried out using the TapeStation D1000 ScreenTape (Agilent Technologies, United States). The paired-end sequencing (2 × 300 bp) was performed using the MiSeq platform (Illumina, United States).

The paired-end sequence reads gained from the MiSeq platform were merged by the FLASH software (Magoč and Salzberg, 2011), and the merged raw sequence reads were trimmed by CD-HIT-OUT (Li et al., 2012). Taxonomic analysis of trimmed sequence reads was carried out using the MG-RAST server (Meyer et al., 2008) with the SILVA SSU databases (Quast et al., 2013). Further, the e-value, percent identity, minimal alignment length, and minimal abundance values were set to 5, 60, 15, and 1, respectively.

### Metatranscriptomic analysis

For metatranscriptomic analysis, RNAs were extracted using a Maxwell 16 LEV simplyRNA tissue kit (Promega, United States) for each sample solution of NM and USM. The extracted RNAs were quantified using the Quant-IT RiboGreen assay kit (Invitrogen, United Kingdom) following the manufacturer’s instructions. RNAs for each sample were independently prepared using the Illumina Stranded Total RNA Library Prep (Illumina, United States). The rRNAs in the total RNAs were removed by Ribo-Zero Plus (Illumina, United States). The remaining mRNA fragments were converted to cDNA using SuperScript II reverse transcriptase (Invitrogen, United Kingdom) with random primers. The second strand cDNA synthesis was carried out using DNA polymerase I with RNase H and dUTP. When the second strand cDNA was synthesized, the fragments underwent end repair, adapter ligation, and adenine bases were added. The products were then purified and enriched with PCR to produce the final cDNA library. The cDNA libraries were quantified using the KAPA library quantification kits for Illumina sequencing platforms (Kapa Biosystems, United States), and qualification was then carried out using the TapeStation D1000 ScreenTape (Agilent Technologies, United States). The paired-end (2 × 150 bp) sequencing was performed using the NovaSeq platform (Illumina, United States).

The annotation for the metatranscriptome of USM and NM was carried out by the MG-RAST server, and the paired-end sequence read files were also merged and trimmed by the server. Viromic analysis was performed using the RefSeq database (Pruitt et al., 2007), and the hierarchical functional analysis was performed using the SEED subsystem database (Overbeek et al., 2005).

### Statistics and visualization

Statistical analyses for the metataxonome and metatranscriptome of USM and NM were done using MicrobiomeAnalyst (Dhariwal et al., 2017). The data normalization was performed for the total sum scaling. The alpha diversities of the metataxonome were calculated using the Phyloseq package (McMurdie and Holmes, 2013) with MicrobiomeAnalyst. A heat map was made using MultiExperiment 4.9.1 (Saeed et al., 2003), and Venn diagrams were created using VENNY 2.1.^1^ Correlation analysis between the metataxonome and metatranscriptome was performed using metaboAnalyst 5.0 (Pang et al., 2021), then bootstrapping for re-sampling was performed 20 times using XLSTAT 2017 (Addinsoft, France).

## Results and discussion

### Sequencing data for metataxonome and metatranscriptome

The kimchi samples were isolated to USM and NM by TFF for the metataxonomic diversity of USM and comparison with NM. The metataxonomic sequencing data for NM and USM were obtained (Table 1). The sequence profiles of the NM and USM were obtained as 61,700,786 and 52,968,776 bp, respectively. After merging and trimming, the total read bases and reads of NM were 854,180 bp and 1,840, and the mean sequence read length was 465 bp, while the GC content was 51%. The total read bases and total reads of USM were 1,279,587 bp and 2,786, and the mean sequence read length was 459 bp, while the GC content was 52%.

**Table 1:** Conditions of sequence reads for each sample by metataxonomic and metatranscriptomic analyses.

Analysis types	Condition indices	USM	NM
Metataxonomic	Raw sequence read bases (bp)	52,968,776	61,700,786
	Trimmed sequence read bases (bp)	1,279,587	854,180
	Total reads	2,786	1,840
	mean sequence read length (bp)	459	465
	GC contents (%)	52	51
Metatranscriptomic	Raw sequence read bases (bp)	2,094,071,926	2,349,741,804
	Trimmed sequence read bases (bp)	47,555,685	219,645,260
	Total reads	197,783	891,252
	Mean sequence read length (bp)	240	246
	GC contents (%)	43	42
	Predicted protein features	36,346	147,923
	Predicted rRNA features	805	2,782

USM, ultra-small microbiome; NM, normal microbiome.

Metatranscriptomic sequencing data for NM and USM were obtained for identification of the potential functional transcriptome of USM and comparison with NM (Table 1). The sequence profiles of the NM and USM were obtained as 2,349,741,804 and 2,094,071,926 bp, respectively. After merging and trimming, the total read bases and reads of NM were 219,645,260 bp and 891,252, and the mean sequence read length was 246 bp. The GC content, predicted protein features, and predicted rRNA features were 42%, 147,923, and 2,782, respectively. The total read bases and total reads of USM were 47,555,685 bp and 197,783, and the mean sequence read length was 240 bp. The GC content, predicted protein features, and predicted rRNA features were 43%, 36,346, and 805, respectively.

### Diversity of the ultra-small microbiome

**Figure 1:**
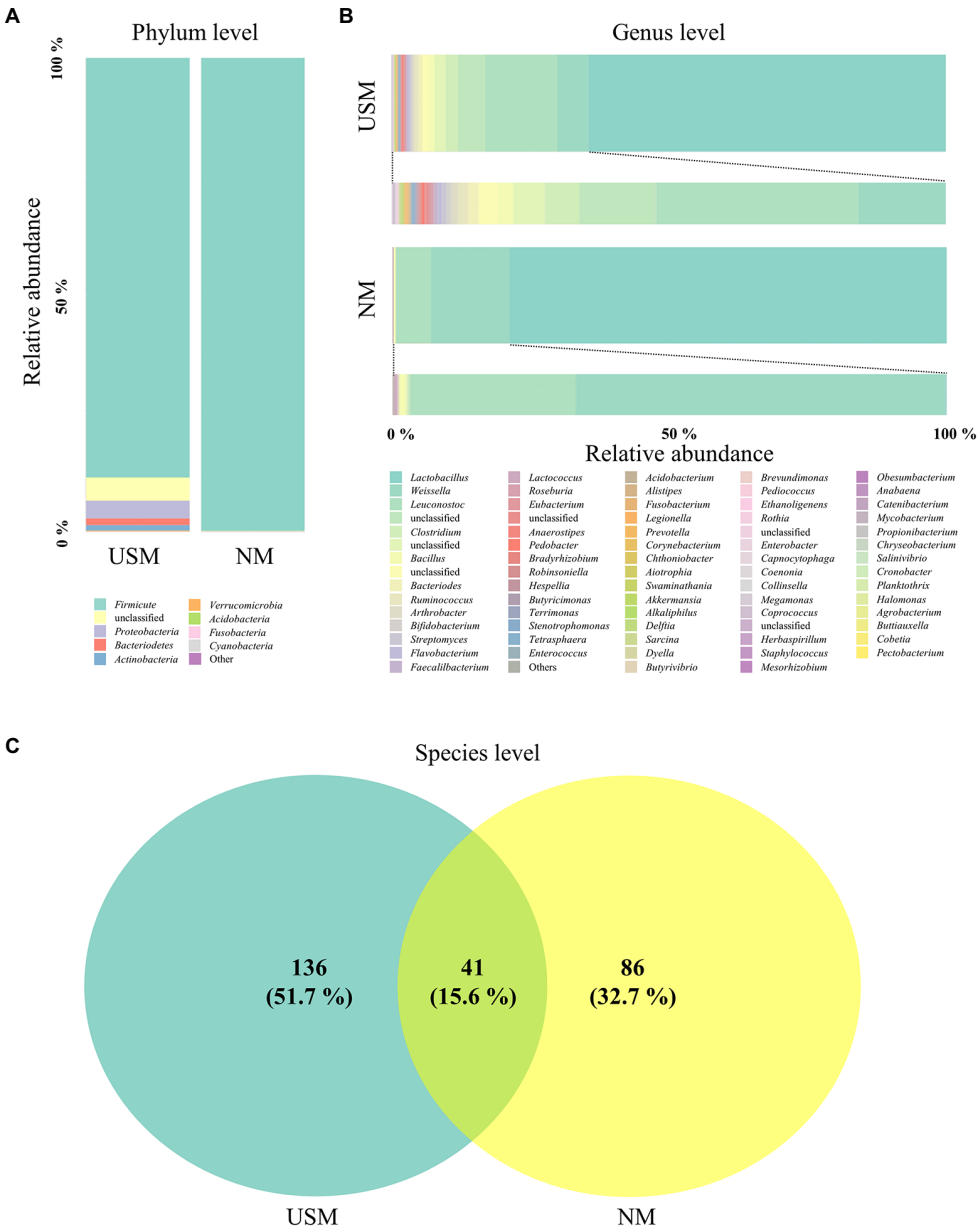
Relative abundance profiling of microbiome analyzed in the USM and NM. Relative abundance profiling of microbial communities was analyzed at the phylum level **(A)** and genus level **(B)**. A Venn diagram confirmed the relationship between samples on the species level **(C)**.

The diversity of the USM in the kimchi sample was compared with NM (Figure 1; Supplementary Table 1) by metataxonomic analysis, and the pH in the kimchi sample was 4.13. The alpha diversity of each taxon is shown in Table 2. The observed operational taxonomic unit (OTU) for USM was higher than that for NM (168 vs. 118). Further, the Chao1, abundance-based coverage estimators (ACE), Shannon, Simpson, and Fisher indices of the USM were 304, 324, 1.64, 0.57, and 20.3, respectively, and those for NM were 188, 194, 0.88, 0.37, and 13.2, respectively.

**Table 2:** The alpha diversities of each sample identified by metataxonomic analysis.

Indices	USM	NM
Observed OTU	168	118
Chao1	304	188
ACE	324	194
Shannon	1.64	0.88
Simpson	0.57	0.37
Fisher	20.3	13.2

OTU, operational taxonomic unit; ACE, abundance-based coverage estimators; USM, ultra-small microbiome; NM, normal microbiome.

The bacterial communities on the phylum level of the USM were dominated by *Firmicutes* (88.48%; Figure 1A), while *Proteobacteria* (3.79%), *Bacteriodetes* (1.42%), and *Actinobacteria* (1.28%) were not so dominant (Figure 1A). The bacterial communities of NM were dominated by *Firmicutes* (99.72%), just like the USM (Figure 1A). The bacterial communities at the genus level of the USM were dominated by *Lactobacillus* (64.52%), while *Leuconostoc* (12.99%), *Weissella* (5.63%), and *Clostridium* (2.21%) were not so dominant (Figure 1B). *Lactobacillus* (78.82%) was dominant in NM, while *Weissella* (14.07%) and *Leuconostoc* (6.30%) were not so dominant (Figure 1B).

At the species level, 41 species overlapped in the USM and NM, while 136 species were found only in USM, and 86 species were found only in NM (Figure 1C; Supplementary Table 2). Most of the overlapping species were those belonging to the genera *Lactobacillus*, *Leuconostoc*, and *Weissella*. Specifically, the relative ratio of *Leuconostoc*, which is known to have many strains that enhance the unique flavor of kimchi in the early stages of fermentation due to its good mannitol production ability (Lee et al., 2020), was significantly higher in the USM than in the NM. It is estimated that the pH of kimchi is 4.13 (Park et al., 2014), but since kimchi reached the ripe stage and *Lactobacillus* occupied the ecosystem, it is estimated that *Leuconostoc* was incorporated into the USM by reducing their size for survival.

The Chao1, ACE, Shannon, Simpson, and Fisher indices indicating alpha diversity were all higher in USM than in NM. These results were identical to those of a previous study (Lee et al., 2022), where the USMs of non-sterilized fermented cabbage varied. From these results, it was deduced that the diversity of the microbiome members below the 0.2 μm size defined by USM was not low. In both USM and NM, *Lactobacillus*, *Leuconostoc*, and *Weissella* belonging to lactic acid bacteria (LAB) were dominant, and this was presumed to be the ripening state with the most *Lactobacillus* spp. in LAB because of the pH of 4.1 in the kimchi sample known for having the high LAB content (Park et al., 2014). In addition, the genus *Akkermansia*, belonging to the phylum *Verrucomicrobia*, was detected only in the USM (Figure 1; Supplementary Table 1). Although *Akkermansia* was detected in a very small amount (0.05%), it was determined that there was a possibility that only USM could be purified and used as a new probiotic since it has just been described as a beneficial bacteria (Cani et al., 2022). However, since *Akkermansia* was not detected in the kimchi USM in the previous study (Lee et al., 2022), we believe that additional research is needed.

Contrary to the previous study (Lee et al., 2022), TM7, called *Saccharibacteria*, was not found in the kimchi sample of this study. It was probably detected in the previous study due to the single-molecule real-time method used, which could read all 16 rRNA genes. In this study, the clonal bridge amplification method that can read only relatively short sequences was used. TM7 may not have been found because only the V3 and V4 regions of the 16S rRNA gene were read by sequencing (Brown et al., 2016; Jeong et al., 2021). Also, some of the members of the USM might have been outer membrane vesicles (OMVs), which are 20 to 200 nm in size and are known to contain partial DNA and RNA (Schwechheimer and Kuehn, 2015; Reimer et al., 2021). Gram-negative bacteria known to produce OMVs (Jan, 2017) have not been detected in USM. However, there is still a possibility that some of the members of the USM identified in this study were OMVs, as there is a study that shows that gram-positive bacteria also emit OMVs (Schwechheimer and Kuehn, 2015).

### Analysis of the potential functional transcriptome

#### Comparison of transcriptomes of USM and NM

**Figure 2:**
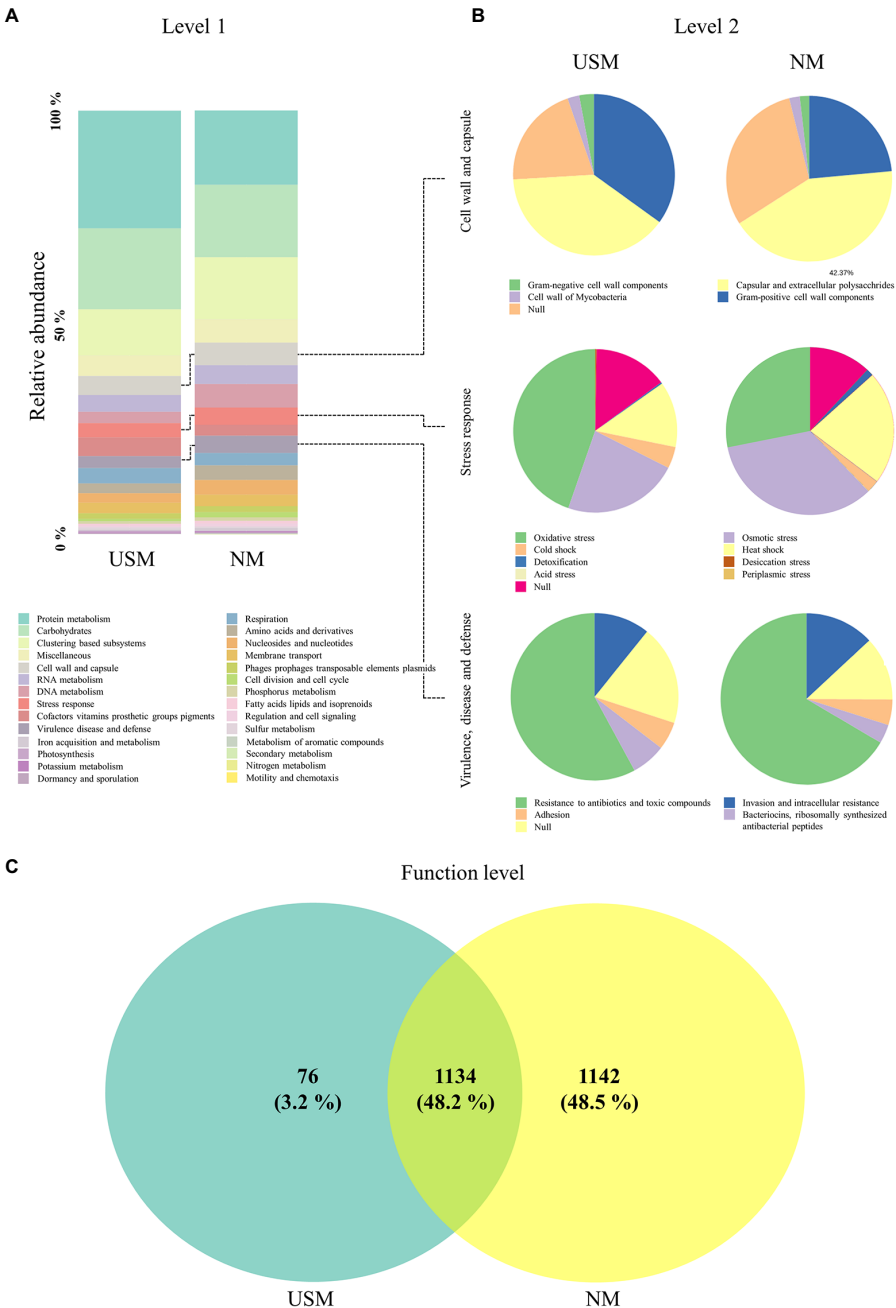
Relative abundance profiling of metatranscriptome analyzed in the USM and NM. The relative abundance profiling of transcripts was analyzed in level 1 **(A)**, and the relative abundance profiling of sub transcripts related to “cell wall and capsule,” “stress response,” and “virulence, disease, and defense” was analyzed in level 2 **(B)**. A Venn diagram confirmed the relationship between samples on the functional level **(C)**.

Metatranscriptome analysis has been used for the investigation of functional and taxonomic dynamics in fermented foods such as hard cheese (Quijada et al., 2022), camembert cheese (Lessard et al., 2014), liquor starter (Huang et al., 2017), pao cai (Xiao et al., 2021), and kimchi (Jung et al., 2013). The transcriptomes of USM in the kimchi sample were compared with those of the NM using metatranscriptomic analysis with the SEED subsystem (Figure 2; Supplementary Table 3). Based on that subsystem in the USM, the metatranscriptome corresponding to level 1 was dominated by transcriptomes related to “protein metabolism” (27.70%), “carbohydrates” (19.11%), and “clustering-based subsystems” (10.93%; Figure 2A). The “cell wall and capsule” (4.52%), ‘cofactors, vitamins, prosthetic groups, pigments” (4.43%), “RNA metabolism” (3.92%), “respiration” (3.62%), “stress response” (3.34%), “virulence, disease and defense” (2.80%), “DNA metabolism” (2.71%), “membrane transport” (2.53%), “amino acids and derivatives” (2.32%), “nucleosides and nucleotides” (2.23%), and “phages, prophages, transposable elements, plasmids” (1.30%) transcriptomes were minorly dominant in the USM (Figure 2A). The transcriptomes of NM were also mainly dominated by “protein metabolism” (17.51%), “carbohydrates” (17.09%), and “clustering-based subsystems” (14.67%; Figure 2A). The “DNA metabolism” (5.57%), “cell wall and capsule” (5.34%), “RNA metabolism” (4.40%), “stress response” (4.05%), “virulence, disease, and defense” (4.05%), “nucleosides and nucleotides” (3.47%), “amino acids and derivatives” (3.45%), “respiration” (2.92%), “membrane transport” (2.73%), “cofactors, vitamins, prosthetic groups, pigments” (2.58%), “phages, prophages, transposable elements, plasmids” (1.39%), and “cell division and cell cycle” (1.23%) transcriptomes were minorly dominant in the NM (Figure 2A).

Compared to NM, the USM showed a relatively higher ratio of transcripts related to ‘protein metabolism.’ Although it is difficult to explain that USM has a higher proportion of protein metabolism-related transcripts than the NM, in general, it is known that the microorganisms that enter the viable-but-nonculturable (VBNC) state in which the cell size decreases (Rahman et al., 1994; Du et al., 2007; Li et al., 2014), aggregate proteins (Bollen et al., 2021) and produce a special protein set (Babin et al., 2017; Mali et al., 2017; Ayrapetyan et al., 2018). It has also been stated that many microorganisms are in the VBNC state in the USM (Lee et al., 2022). Therefore, the relative increase in protein metabolism-related transcripts in the USM than in NM was presumed to be related to protein aggregation and production of specific protein sets in the VBNC state. Transcripts of other categories classified at level 1 did not differ relatively much in the USM and NM.

#### Transcriptomes related to “cell wall and capsule”

In the USM, the level 2 transcriptome associated with the “cell wall and capsule” was occupied by RNA transcripts associated with “capsular and extracellular polysaccharides” (39.10%), “Gram-positive cell wall components” (34.97%), “Gram-negative cell wall components” (2.95%), and “cell wall of mycobacteria” (2.35%; Figure 2B). In the NM, it was also occupied by RNA transcripts associated with “capsular and extracellular polysaccharides” (42.37%), “Gram-positive cell wall components” (23.55%), “cell wall of mycobacteria” (2.06%), and “Gram-negative cell wall components” (1.82%; Figure 2B).

#### Transcriptomes related to “stress response”

In the USM, the level 2 transcriptome associated with the “stress response” was occupied by transcripts associated with “oxidative stress” (44.7%), “osmotic stress” (22.9%), “heat shock” (13.0%), and “cold shock” (4.3%; Figure 2B). In the NM, it was occupied by transcripts associated with “osmotic stress” (34.1%), “oxidative stress” (28.1%), “heat shock” (22.0%), “cold shock” (2.4%), and “detoxification” (1.4%; Figure 2B).

#### Transcriptomes related to “virulence, disease, and defense”

In the USM, the level 2 transcriptome associated with “virulence, disease and defense” was occupied by transcripts associated with “resistance to antibiotics and toxic compounds” (57.9%), “invasion and intracellular resistance” (10.8%), “bacteriocins (ribosomally-synthesized antibacterial peptides)” (6.6%), and “adhesion” (5.4%; Figure 2B). In the NM, it was occupied by transcripts associated with “resistance to antibiotics and toxic compounds” (66.5%), “invasion and intracellular resistance” (13.0%), “adhesion” (4.8%), and “bacteriocins (ribosomally-synthesized antibacterial peptides)” (3.6%; Figure 2B).

#### Transcripts at the functional level

The transcripts at the functional level of the USM and NM overlapped (48.2%), and for the USM, only 3.2% had unique transcripts, while 37.9% of the NM had unique transcripts. However, the abundance of transcripts was not considered (Figure 2C; Supplementary Table 3).

#### Comparison of transcriptome related to survival

Since the formation of USM is thought to be related to survival, the lower transcripts were identified in the functional classification categories of level 1 “cell wall and capsule,” “stress response,” and “virulence and defense” and compared with the NM. In the “cell wall and capsule,” the transcripts related to “Gram-negative cell wall components” and “Gram-positive cell wall components” had a relatively higher proportion in the USM than in the NM. It was possible that as the cell size became small enough to belong to the USM, the ratio of the transcripts related to the cell wall components became relatively high. The transcripts related to “oxidative stress” in “stress response” had a relatively higher proportion in the USM than in the NM. Some studies (Desnues et al., 2003; Yoon et al., 2020) have shown that many oxidative stress-related proteins are expressed since VBNC is formed due to oxidative stress. Therefore, it was deduced that there were more bacteria in the VBNC state of the USM than that of the NM. In contrast, the relative proportion of transcripts related to “virulence, disease, and defense” category was lower in the USM (2.80%) than in the NM (4.05%). The ratio of transcripts related to “resistance to antibiotics and toxic compounds” in “virulence, disease, and defense” was also lower in the USM than in the NM. In general, microorganisms in the VBNC state cannot attach to enterocytes or plastics and cannot form biofilms (Pruzzo et al., 2003; Lleo et al., 2007) as they may lose their infectivity or become toxic (Oliver and Bockian, 1995; Lindbäck et al., 2010). From these previous studies, if a member contains a lot of bacteria in the VBNC state, the expression of transcripts related to “virulence, disease, and defense” becomes low. Therefore, it was presumed that there are many bacteria in the VBNC state in the members of the USM. Furthermore, some bacteria in the USM may be OMVs because the size of OMVs with luminal DNA is small (100–300 nm; Jan, 2017). However, transcriptome transcribed into mRNA was thought to be evidence that USM is alive and exists (Li et al., 2014). Since the types of transcripts identified in the USM at the functional level were less than those in NM (Figure 2C), they were thought to minimize the types of proteins expressed in the USM.

#### RNA virome

**Figure 3:**
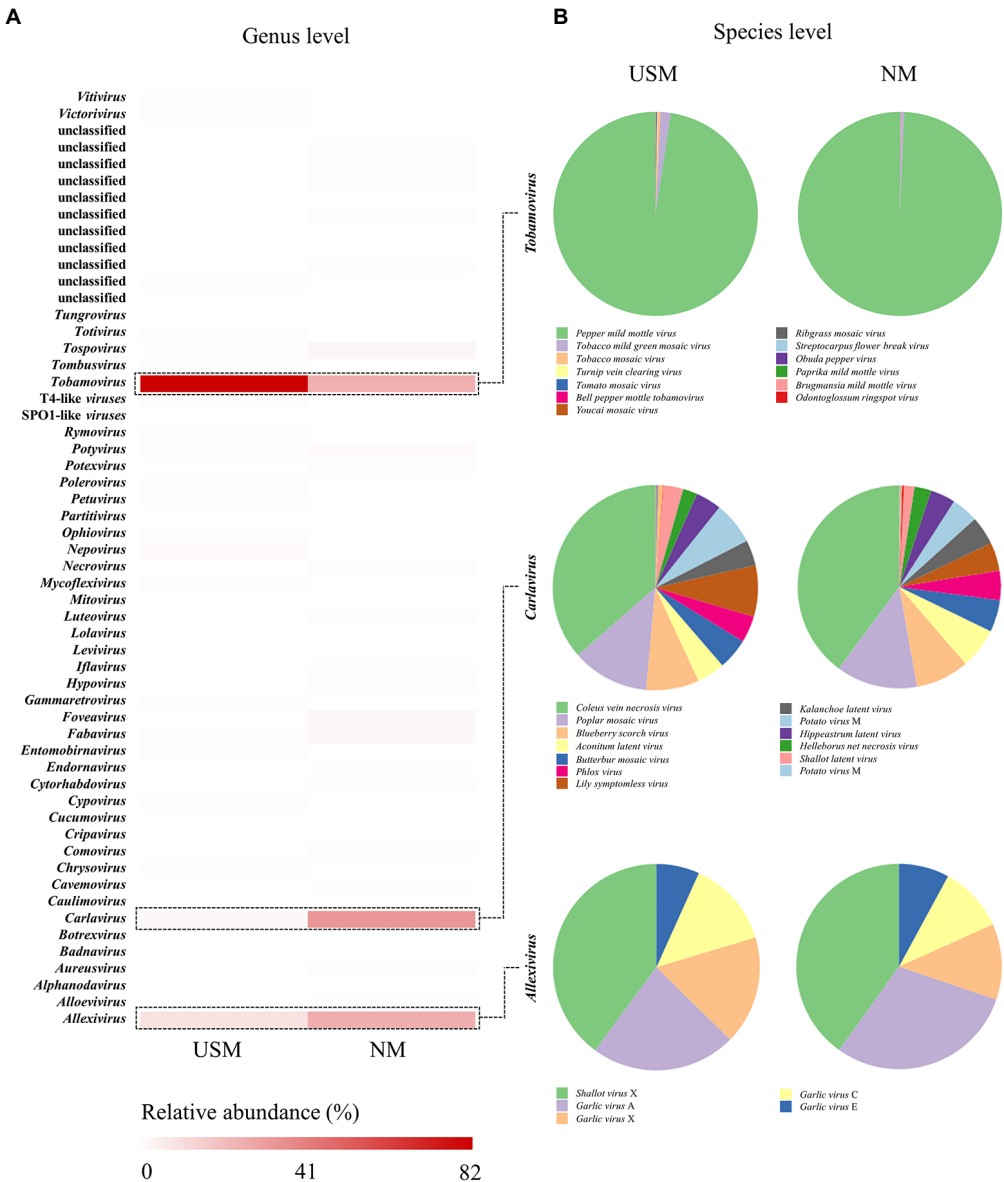
Relative abundance profiling of RNA virome analyzed in USM and NM. Relative abundance profiling of RNA virus in the genus level was analyzed using a heat map **(A)**, and the red color intensities of panels are proportional to the operational taxonomic unit (OTU) abundance (max. 82%). Relative abundance profiling of species levels related to *Tobamovirus*, *Carlavirus*, and *Allexivirus* was analyzed using pie charts **(B)**.

In addition, the RNA virome of USM in the kimchi sample was compared with NM by metatranscriptomic analysis using RefSeq (Figure 3; Supplementary Table 4) by the Virus Metadata Resource (VMR) based on the International Committee on Taxonomy of Viruses (ICTV).^2^ The RNA viral communities on the genus level of the USM were mainly dominated by *Tobamovirus* (82.09%) and *Allexivirus* (9.35%). *Carlavirus* (2.63%) and *Nepovirus* (1.93%) were minorly dominant in the USM (Figure 3A). The RNA viral communities on the genus level of the NM were mainly dominated by *Carlavirus* (33.98%), *Allexivirus* (26.01%), and *Tobamovirus* (25.63%). Those with minor dominance in the NM were *Tospovirus* (2.99%), *Fabavirus* (2.84%), *Foveavirus* (2.58%), and Potyvirus (1.77%; Figure 3A). Further, at the species level, *pepper mild mottle virus* (PMMoV) was dominant in both the USM (80.10%) and NM (25.45%; Figure 3B).

Viruses such as *Tobamovirus*, *Carlavirus*, and *Allexivirus*, which are mainly parasitic on plants (Hulo et al., 2011), accounted for the majority. Since the samples in this study were mostly kimchi made of plant materials, the USM and NM were thought to have a high ratio of plant viruses (RNA viruses). However, PMMoV belonging to *Tobamovirus* was highly dominant in the USM and NM. In a study by Kim et al. (2014), it was found that most of the plant RNA viruses of kimchi were PMMoV, which is consistent with our results. Moreover, the reason for the high ratio of PMMoV as the major pathogenic virus of plants in the genus *Capsicum* (Jarret et al., 2008) in the USM and NM was presumed to be due to the chili powder used in kimchi. Although the RNA virome was identified through metatranscriptome analysis in this study, it could not be related to the metataxonome. In addition, although prophage forms exist in bacterial cells, free RNA viruses are also presumed to exist, so it was judged that the difference between the USM and the NM might be a coincidence.

### Correlation between metataxonome and metatranscriptome identified USM and NM

**Figure 4:**
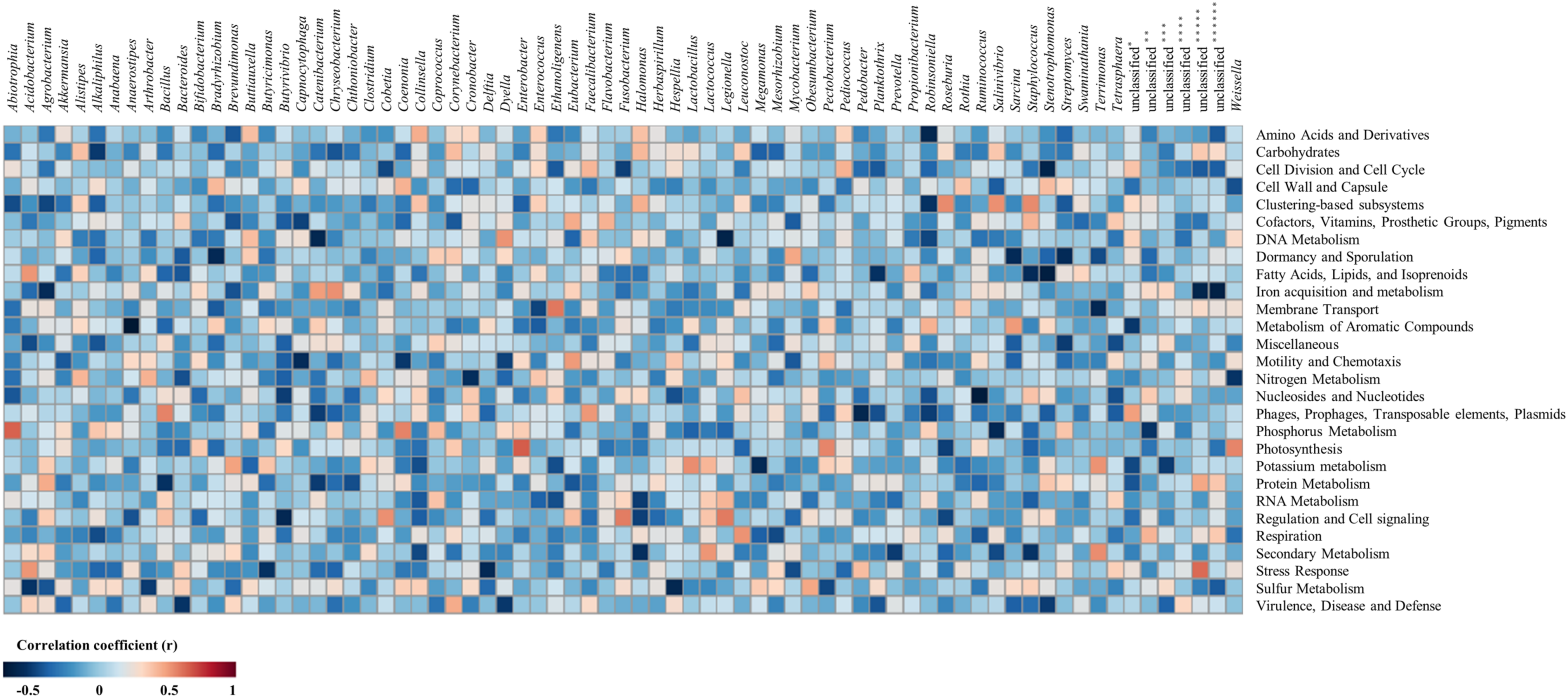
Heatmap for correlation between metataxonome and metatranscriptome. The metataxonome was at the genus level, and the metatranscriptome was at level 1. The re-sampling by bootstrapping was performed 20 times. ^*^, unclassified bacteria; ^**^, unclassified bacteria derived from *Betaproteobacteria*; ^***^, unclassified bacteria derived from *Clostridiales*; ^****^, unclassified bacteria derived from *Erysipelotrichaceae*; ^*****^, unclassified bacteria derived from *Gammaproteobacteria*; ^******^, unclassified bacteria derived from *Verrucomicrobia* subdivision 3.

The correlation analysis between metataxonome and metatranscriptome was expressed as a heatmap (Figure 4; Supplementary Table 5). Transcripts related to “virulence, disease, and defense” was negatively correlated with *Bacteroides* (*r* = −0.51) at the genus level. Transcripts related to “stress response” were negatively correlated with *Butyricimonas* (*r* = −0.52) and *Delftia* (*r* = −0.57). Both positive and negative correlation coefficients did not exceed 0.5 large in transcripts related to “cell wall and capsule,” so it was considered that the correlation with bacteria at the genus level was not.

Transcripts of categories of “virulence, disease and defense” and “stress response,” which are judged to be related to survival, were negatively correlated with bacteria. Genus *Bacteroides* negatively correlated (r = −0.51) with “virulence, disease and defense” was found only in USM (Figure 1B; Supplementary Table 1), and it can be seen that “virulence, disease, and defense” also expressed relatively low USM compared to NM (Figure 2A). Genera *Butyricimonas* and *Delftia* negatively correlated (*r* = −0.52 and *r* = −0.57) with “stress response” were found only in USM (Figure 1B; Supplementary Table 1), respectively. It can also be seen that “stress response” expressed relatively low USM compared to NM (Figure 2A). Bacteria in the metataxonome that were correlated with “cell wall and capsule” did not appear.

Transcripts related to “sulfur metabolism” were negatively correlated with *Acidobacterium* (*r* = −0.51) and *Hespellia* (*r* = −0.57). Transcripts related to “secondary metabolism” were positively correlated with *Lactococcus* (*r* = 0.52) and *Terrimonas* (*r* = 0.55), were negatively correlated with *Halomonas* (*r* = −0.53) and *Staphylococcus* (*r* = −0.51). Transcripts related to “respiration” were positively correlated with *Leuconostoc* (*r* = 0.53). Transcripts related to “regulation and cell signaling” were positively correlated with *Cobetia* (*r* = 0.54), *Fusobacterium* (*r* = 0.58) and *Legionella* (*r* = 0.59), were negatively correlated with *Butyrivibrio* (*r* = −0.52). Transcripts related to “protein metabolism” were negatively correlated with *Bacillus* (*r* = −0.52). Transcripts related to “potassium metabolism” were positively correlated with *Lactobacillus* (*r* = 0.54), and were negatively correlated with *Megamonas* (*r* = −0.57). Transcripts related to “photosynthesis” were positively correlated with *Enterobacter* (*r* = 0.67), *Pectobacterium* (*r* = 0.57) and *Weissella* (*r* = 0.57). Transcripts related to “phosphorus metabolism” were positively correlated with *Abiotrophia* (*r* = 0.65) and *Coenonia* (*r* = 0.57), were negatively correlated with *Salinivibrio* (*r* = −0.57). Transcripts related to “phages, prophages, transposable elements, plasmids” were positively correlated with *Bacillus* (*r* = 0.57) and *Faecalibacterium* (*r* = 0.52), were negatively correlated with *Pedobacter* (*r* = −0.59). Transcripts related to “nucleosides and nucleotides” were negatively correlated with *Ruminococcus* (*r* = −0.66). Transcripts related to “nitrogen metabolism” were negatively correlated with *Cronobacter* (*r* = −0.54) and *Weissella* (*r* = −0.51). Transcripts related to “motility and chemotaxis” were positively correlated with *Eubacterium* (*r* = 0.52), and were negatively correlated with *Capnocytophaga* (*r* = −0.55). Transcripts related to “metabolism of aromatic compounds” were positively correlated with *Sarcina* (*r* = 0.51). Transcripts related to “Membrane Transport” were positively correlated with *Ethanoligenens* (*r* = 0.60), and were negatively correlated with *Terrimonas* (*r* = −0.57). Transcripts related to “iron acquisition and metabolism” were positively correlated with *Catenibacterium* (*r* = 0.50) and Chryseobacterium (*r* = 0.54), and were negatively correlated with *Agrobacterium* (*r* = −0.55). Transcripts related to “fatty acids, lipids, and isoprenoids” were positively correlated with *Acidobacterium* (*r* = 0.53), and were negatively correlated with *Planktothrix* (*r* = −0.51), *Staphylococcus* (*r* = −0.55) and *Stenotrophomonas* (*r* = −0.64). Transcripts related to “dormancy and sporulation” were negatively correlated with *Bradyrhizobium* (*r* = −0.57), *Sarcina* (*r* = −0.56) and *Streptomyces* (*r* = −0.56). Transcripts related to “DNA metabolism” were positively correlated with *Dyella* (*r* = 0.53), and were negatively correlated with *Catenibacterium* (*r* = −0.57) and *Legionella* (*r* = −0.60). Transcripts related to “clustering-based subsystems” were positively correlated with *Robinsoniella* (*r* = 0.55), *Salinivibrio* (*r* = 0.54) and *Staphylococcus* (*r* = 0.56). Transcripts related to “cell division and cell cycle” were negatively correlated with *Stenotrophomonas* (*r* = −0.60). Transcripts related to “amino acids and derivatives” were negatively correlated with *Robinsoniella* (*r* = −0.58). In addition, both positive and negative correlation coefficients did not exceed 0.5 in transcripts related to “RNA metabolism,” “cofactors, vitamins, prosthetic groups, pigments” and “carbohydrates,” so it was considered that the correlation with bacteria at genus level was not.

Since “sulfur metabolism” and “dormancy and sporulation” only showed a negative correlation with bacteria (genera *Acidobacterium* and *Hespellia* for “sulfur metabolism”; genera *Bradyrhizobium*, *Sarcina,* and *Streptomyces* for “dormancy and sporulation”) belonging to USM, there is a possibility that the expression level of A-related transcripts is low in USM. On the other hand, “membrane transport” showed a positive correlation only with bacteria (genera *Ethanoligenens* and *Terrimonas*) belonging to USM, and it is possible that the expression level of transcripts related to “membrane transport” in USM is relatively higher than that of NM. Also, there was no relationship between metataxonome and metatranscriptome with positive or negative correlation coefficients greater than 0.8, but there were relationships with positive or negative correlation coefficients greater than 0.5. Therefore, it is thought that the metataxonome and metatranscriptome influence each other to some extent. However, only limited samples were obtained due to the difficulty of pretreatment. To overcome this, since the correlation was analyzed with samples obtained by bootstrapping, more research is needed to obtain clearer results.

## Conclusion

In this study, we compared the USM metataxonome and metatranscriptome of kimchi, passed through a filter with pores of 0.2 μm or less using TFF, to those of NM. From our results, the USM and NM of kimchi showed different microbial diversity, and transcripts were also expressed differently. Further, unlike the NM, the metatranscriptome of the USM showed some of the characteristics of VBNC confirmed in previous studies, so it was estimated that there were many bacteria in the VBNC state of the USM. While the results of the metatranscriptomic and metataxonomic analyses of the USM can be controversial, it should always be considered that a small number of microorganisms trying to survive in a colony of other microbes have also formed their separate colonies too. Based on these analyses, viruses and transcriptomes of the UMB, OMV, and bacteria of VBNC states, which are expected to be mixed in the USM, were studied. This is considered a stepping stone that can contribute greatly to related research in the future.

## Data availability statement

The datasets presented in this study can be found in online repositories. The names of the repository/repositories and accession number(s) can be found at: https://www.ncbi.nlm.nih.gov/, SRR21133393; https://www.ncbi.nlm.nih.gov/, SRR21133394; https://www.ncbi.nlm.nih.gov/, SRR21133488; and https://www.ncbi.nlm.nih.gov/, SRR21133489.

## Author contributions

H-WL: writing—original draft, investigation, data curation, conceptualization, software, methodology, and funding acquisition. S-RY: writing—review and editing, and investigation. Y-MD: investigation and data curation. J-HY: investigation, methodology, and software. HJ and K-NK: methodology and data curation. J-HH: validation, resources, writing—review and editing, funding acquisition, and supervision. J-WB: writing—review and editing, data curation, and supervision. All authors contributed to the article and approved the submitted version.

## Funding

This work was supported by the National Research Foundation of Korea (NRF-2019R1F1A1061368) and the World Institute of Kimchi (KE2202-2).

## Conflict of interest

The authors declare that the research was conducted in the absence of any commercial or financial relationships that could be construed as a potential conflict of interest.

## Publisher’s note

All claims expressed in this article are solely those of the authors and do not necessarily represent those of their affiliated organizations, or those of the publisher, the editors and the reviewers. Any product that may be evaluated in this article, or claim that may be made by its manufacturer, is not guaranteed or endorsed by the publisher.

## Footnotes

^1^ https://bioinfogp.cnb.csic.es/tools/venny/

^2^ https://ictv.global/
